# Indoor air quality at school and students’ performance: Recommendations of the UNESCO Chair on Health Education and Sustainable Development & the Italian Society of Environmental Medicine (SIMA)

**DOI:** 10.34172/hpp.2020.29

**Published:** 2020-07-12

**Authors:** Manuela Pulimeno, Prisco Piscitelli, Salvatore Colazzo, Annamaria Colao, Alessandro Miani

**Affiliations:** ^1^UNESCO Chair on Health Education and Sustainable Development, Naples, Italy; ^2^Doctorate in Human Relations Sciences, University of Bari “Aldo Moro”, Bari, Italy; ^3^Italian Society of Environmental Medicine (SIMA), Milan, Italy; ^4^Department of History, Society and Human Studies, University of Salento, Lecce, Italy; ^5^Department of Clinical Medicine and Surgery, Federico II University School of Medicine, Naples, Italy

**Keywords:** Indoor air quality, School, Students, Performance, Health, COVID-19

## Abstract

The issue of indoor air quality (IAQ) concerns 64 million students across Europe, but it is still a neglected topic, although it impacts both their health and learning outcomes. Classroommicroclimate is the first key factor determining a healthy or unhealthy school environment, and it is influenced by ventilation, temperature and humidity rate. Classrooms are usually crowded, overheated and poorly ventilated, thus resulting in possible increases of carbon dioxide (CO_2_), that can cause several problems when its concentrations exceed the value of 0.15 percentage volume of CO_2_ (1500 ppm) or even at lower levels (1000 ppm). CO_2_ can also arise from outside the school, being widely produced by the combustion of fossils or road traffic. Anthropogenic activities are responsible for the emission of nitrogen dioxide (NO_2_) and polycyclic aromatic hydrocarbons(PAH) too, which represent other possible external contaminants potentially impairing IAQ. Furtherdangerous exposures for students’ health are those related to natural emission of gas Radon, which typically accumulates in poorly ventilated classrooms, and volatile organic compounds (VOCs, released by building materials, paints, furnishings, detergents), while chemicals substances (i.e.cyanoacrylate, lead, cadmium, nickel) might be contained in school materials. Finally, particulate matter (PM2.5 and PM10) originating from road traffic, domestic heating or industrial activities represent additional possible contaminants impacting schools’ air quality. Poor IAQ might result in mild adverse events (i.e. headaches, nausea etc.) or cause respiratory problems. More frequently, IAQ affects students’ attention and their school performances, as widely documented by many studies. Standardized tests administered to pupils exposed to poor IAQ (to assess reading and mathematical abilities) systematically result in worse outcomes compared to students staying in healthy classroom environments. In this paper, we present recommendations of UNESCO Chair on Health Education and Sustainable Development and Italian Society of Environmental Medicine(SIMA) to ensure an optimal IAQ at school, including some post-COVID-19 issues.

## Indoor air quality at school

What kind of air do young people breathe at school and how it affects their academic performance? The question of indoor air quality (IAQ) concerns 64 million students (and 4.5 million teachers) across Europe, but it is still a neglected topic, although it significantly impacts both their global wellbeing and learning achievements.^1^ Basic factors determining a healthy or unhealthy microclimate at school are: ventilation, temperature and humidity rate.^2-6^ Classrooms are usually crowded, overheated and poorly ventilated, or even contaminated by pollutants originating both from inside the school and outside (road traffic, domestic heating, intensive agriculture or industrial activities etc).^1,2,7^

Concerning overcrowding, available evidence shows that the number of students is directly and significantly associated with higher concentrations of carbon dioxide (CO_2_) in the classroom (up to +25%).^6^ Normal breathing of a child aged 7-9 years old generates 14 L of CO_2_ per hour, which is 50% lower than the amount produced by a teenager (in conditions of moderate physical activity, a 15 years old student can release up to 85 L of CO_2_ per hour).^6^CO_2_ makes classrooms uncomfortable. International standards (such as the DIN 1946 UNI EN standard) tolerate a maximum value of 0.15 percentage volume of CO_2_ (1500 parts per million, ppm), but – depending on the age – the individual tolerable limit values can be reached earlier (from 1000 ppm of CO_2_).^6^ When this limit is overcome, air becomes stagnant and could cause headaches, tachycardia, nausea, memory disturbances, lack of concentration, blurred vision, sweating, restlessness, vomiting, flushed skin, and even panic attacks.^7^ CO_2_ can arise also from outside the school, being widely produced by combustion of fossils or road traffic and other anthropogenic activities, which are responsible for the emissions of nitrogen dioxide (NO2), as well as polycyclic aromatic hydrocarbons (PAH; i.e. benzopyrene).^8-11^ Indeed, NO2 represents another possible contaminant originating from external sources that can impair school IAQ and cause respiratory problems in schoolchildren (i.e. asthma exacerbations, increased susceptibility to viral infections etc).^12,13^

In addition to the residual asbestos manufactures (that should be removed with priority by authorized companies), a further potential source of dangerous exposures for students’ health is the natural emission of gas Radon coming from underground cavities, which typically accumulates in poorly ventilated classrooms and can affect lung function in case of chronic exposures.^14^ Moreover, volatile organic compounds (VOCs) - such as formaldehyde, toluene, benzene – can be released by building materials, paints, furnishings, and detergents.^15-17^ Like Radon, many VOCs are not perceptible by smell but can negatively affect health even at concentrations lower than 3 μg/m^3^. Exposures to these kind of compounds at school may cause dizziness, headache, allergies, eye/nose/throat irritation, dyspnoea, fatigue and deficit of attention.^18^

Particulate matters (PM2.5 and PM10) represent additional pollutants that can impair IAQ at school.^19^Actually, in a study published by Branis and colleagues, it was found that IAQ in the classrooms is influenced not only by internal factors such as crowding and microclimate (poor ventilation and overheating), as already mentioned, but also by particulate matter attributable to road traffic emissions.^20^ This research demonstrated that indoor concentrations of PM10 can reach average levels of 40 μg/m^3^, with peaks even higher than 75 μg/m^3^ (daily average limit 50 μg/m^3^).^20^ The European Commission has carried out in 2015 a specific survey (*SInPHONiE - Schools Indoor Pollution and Health Observatory Network in Europe* ) to assess air quality in 114 primary schools (5575 students) across 23 EU countries. According to this study, about 85% of students are exposed to PM2.5 and PM10 concentrations higher than those considered safe by the World Health Organization in 2005 for the prevention of cardio-pulmonary diseases.^21^

## School indoor air quality and students’ performance

A number of researches have documented the association between IAQ and students’ school performance.^22-26^Haverinen-Shaughnessy and colleagues – in a study involving 100 schools in the United States – demonstrated that classroom ventilation rates are directly associated with students’ academic achievements, and that measurable progresses in maths and reading (assessed through standardized tests) may be observed when improving IAQ in the classrooms.^27^ These conclusions are confirmed by specific systematic reviews and by a big cohort study performed on more than 8000 children in UK.^28-30^Hutter et al examined a total of 436 schoolchildren in Austrian urban areas and observed reduced cognitive performance in those classrooms where higher concentrations of particulate matter (PM10, PM2.5) and CO_2_ were measured.^31^

In a cohort study involving 60 Scottish schools, CO_2_ levels were associated to lower average annual attendance and worse individual test scores for reading, writing and arithmetic – even when adjusted for socio-economic status and number of students per class.^32^ Increased CO_2_ levels were found to reduce short-term students’ attention performance in experimental studies with 20 cluster-randomized classrooms in Germany and in 51 primary schools in Portugal.^33,34^

The relationship between air pollution generated from road traffic and impaired IAQ, as well as the related consequences on school performance were assessed in a Spanish cohort study published by Sunyer and colleagues on students attending 19 schools in Barcelona. The authors of the research showed a better trend (up to + 13%) in cognitive development indicators – such as attention and memorization capacity – in those schools with the lowest levels of traffic-related ultrafine particulate, carbon particles and NO2.^35^ The correlation between IAQ linked to road traffic and the cognitive performance of school-aged children was then confirmed by an annual extension of the same study in 39 schools.^36^ However, according to other authors, classroom internal factors negatively affecting IAQ seem to have a higher impact than pollutants coming from outside.^3,37,38^

Some remedies have been proposed to improve IAQ in the classroom, and reduce the impact of air pollutants on students’ health and on their academic achievements. Polidori and colleagues have demonstrated – in a study carried out in 2013 on 9 classes of Californian schools – that it is possible to significantly improve IAQ in the classrooms thanks to high performance filter systems for air conditioners integrated with air purifiers, which can generate a reduction of 90%-96% in the concentrations of ultrafine particulate matter (PM<1), fine particulate (PM 2.5/PM10), and carbon particles (the dangerous “black carbon”).^39^ Other studies published on the same topic seem to confirm these positive results.^40-45^ Concerning expected health benefits, Martenies and Batterman have showed the possibility of reducing the incidence of asthma from 16% to 13% among children by simply applying filters for PM 2.5 in the classrooms.^46^

A study performed by Gilraine – about the ability of air filters to remove indoor pollutants and consequently improve students’ academic achievements – has been recently published by the Annenberg Institute for School Reform at Brown University of Providence in Rhode Island (USA).^47^ This work confirms the increased students’ school performance just one year after the installation of air filters in the schools of Porter Ranch in San Fernando Valley (Southern California), where these filters have been placed by the local authorities in 2016 due to an alarm concerning methane leaks from a large Californian gas pipeline. These long-term data confirm what emerged from similar assessments previously performed on daily basis by Sefi Roth at the London School of Economics.^48^

## Recommendations proposed by the UNESCO Chair on Health Education and Sustainable Development & SIMA

As we have enough evidence that IAQ in the classroom impacts both students’ health and their learning achievements, it is very important to adopt all the necessary measures to ensure an optimal IAQ at school. The U.S. Environmental Protection Agency (EPA) has already established since many years specific standards and a stable working team on IAQ topics, setting up a Reference Guide for Indoor Air Quality in Schools designed for the IAQ coordinators (usually appointed in US schools or at least at district level) about the most common issues related to IAQ management.^49^ In 2020, the issue of IAQ in school setting has becomes particularly relevant due to the COVID-19 emergency.

Based on the available evidence and consistently with the WHO^1^ and European Commission Guidelines for Indoor Air Quality (developed in Italy by the Governmental Agency ISPRA and the National Institute of Health ISS), the UNESCO Chair on Health Education and Sustainable Development and the Italian Society of Environmental Medicine (SIMA) propose the following recommendations that include useful suggestions to cope with issues related to COVID-19 pandemic:

- Classroom overcrowding should be avoided;
- Teachers and school staff should be informed that poor IAQ impacts both pupils’ health and their academic performance;
- Personal hygiene of the students should be encouraged, in order to prevent they bring at school potential sources of contaminants (animal hair on pupils’ clothes, mould or other dirty stuffs under the shoes etc);
- Classrooms must be adequately ventilated before the beginning of the lessons and during each break (possibly overcoming organizational or logistic problems). To compute the maximum time interval that can elapse between each change of air (opening windows), several factors must be taken into account such as the volume of the room, the age and the number of the children in the classroom, the number of hours spent and the kind of activities that take place in the room. It must be noticed that folding windows do not supply all areas of the room with fresh air, so that windows and doors must be simultaneously opened in order to generate a complete air exchange;
- Surfaces of worktables and chairs, as well as teaching materials, should be cleaned with damp cloths every morning. It must be taken into account that there is evidence that more children develop allergic symptoms if classrooms are cleaned only in the evening, and especially if vacuum cleaners are not used.^21^ Particular attention must be paid to removing mould or dampness. Moreover, when cleaning the classrooms, windows should be kept open or ventilation systems should be turned on, in order to effectively reduce dust, particulate and radon concentrations.
- Equipment such as photocopiers and printers that produce particulates and VOCs must be placed outside the classroom;
- School materials containing chemicals or toxic substances (i.e. cyanoacrylate, lead, cadmium, nickel etc) should be avoided;
- The adoption of protocols and measures for monitoring IAQ should be fostered in every school;
- School buildings should be surrounded by green spaces and trees wherever possible,^50^ in order to create a “green barrier” towards external sources of pollutants. Moreover, specific plants, that can adsorb different indoor contaminants (i.e. formaldehyde, toluene, benzopyrene etc.) by acting as natural filters, can be placed inside the school in order to improve air quality.^51-53^
- Thermostats should be installed in each classroom in order to monitor the temperature (and possibly also the humidity rate that should ideally be 45%-55%) and avoid overheating or dry air. The installation of dehumidifiers (with adequate maintenance of the filters) should also be considered, if necessary.
- Cigarette smoking must be avoided inside the schools (in classrooms, bathrooms, halls) and surrounding spaces, even where national laws do not forbid it expressly;
- School buildings renovation should be fostered whenever possible, giving priority to asbestos removal (only by authorized companies),^54^ and paying attention to the use of water resistant paints or furniture that can release dangerous molecules (i.e. toluene, benzene, xylenes, ethylbenzene), as well as to potential sources of life-threatening compounds such as carbon monoxide (i.e. heating systems, that must undergo annual maintenance and be always placed in boiler rooms separate from the main school building)^6,55^;
- Annual monitoring campaign to assess average indoor concentration of Radon and PM 2.5/PM10, should be performed under the supervision of experts or in cooperation with the Regional/Local Health Authorities;
- The installation of high performance filter systems for air conditioners together with air purifiers (paying attention to the adequate maintenance of the filters on monthly basis) that neutralize fine dust should be considered if the outside air has potential pollution problems, at least in those schools situated nearby traffic-intensive roads or traffic lights, ports, railroads, airports or industrial areas and farms^56^;
- The installation of air decontamination filters, able to eradicate micro-organisms and viruses (up to 0.1 micron size), already used in different public and private settings, could be considered in the perspective of school re-opening after the COVID-19 emergency.^57^

## Ethical approval

Not applicable.

## Competing interests

All authors declare no competing interests.

## Funding

No external funding.

## Authors’ contributions

MP, PP, SC, AC, and AM conceived, wrote, prepared and revised the manuscript.

## Acknowledgments

The UNESCO Chair on Health Education and Sustainable Development and the Italian Society of Environmental Medicine are grateful to the UNESCO Assistant Director-General for Education Dr. Stefania Giannini and her staff. For any other detail on UNESCO Chair objectives, programs and staff organization, please visit https://www.unescochairnapoli.it.
